# Fabrication of Highly Ordered Macropore Arrays in p-Type Silicon by Electrochemical Etching: Effect of Wafer Resistivity and Other Etching Parameters

**DOI:** 10.3390/mi16020154

**Published:** 2025-01-28

**Authors:** Jing Zhang, Faqiang Zhang, Mingsheng Ma, Zhifu Liu

**Affiliations:** 1 CAS Key Laboratory of Inorganic Functional Materials and Devices, Shanghai Institute of Ceramics, Chinese Academy of Sciences, Shanghai 201899, China; 2 College of Materials Science and Opto-Electronic Technology, University of Chinese Academy of Sciences, Beijing 100049, China

**Keywords:** ordered macropore arrays, electrochemical etching, silicon resistivity, pore morphology

## Abstract

Macroporous silicon is a promising substrate in the field of optics, electronics, etc. In this paper, highly ordered macropore arrays were fabricated in p-type silicon wafers by electrochemical etching using a double-tank cell. The effect of the silicon resistivity, etching voltage and etching time on the pore morphology was investigated and the influence mechanism was analyzed. The pore diameter would decrease with the increase in the silicon resistivity and the decrease in the etching voltage, due to the increase in the space charge region width (SCRL). The pore depth would increase with the resistivity and the voltage. However, too high resistivity would cause insufficiency at the pore tips and too high voltage would cause pore splitting, which may cause a decrease in the pore depth. Then, the aspect ratio of 21 can be obtained on the silicon wafer with a resistivity of 50–80 Ω·cm at the etching voltage of 5 V with a maximum etching rate of about 0.92 μm/min.

## 1. Introduction

Macroporous silicon has received much attention due to its controllable shape and size, compatibility with Si technology and promising potential in the applications of chemical sensors [1], photodetectors [2,3], optical devices [4,5], biomedicine [6], capacitors [7,8], solar cells [9] and so on. Although there are several methods to fabricate macropore arrays, such as reactive ion etching and metal-assisted etching, electrochemical etching is widely studied because of its low-cost equipment, controllable process and less metal contamination [10]. Macropore fabrication was first reported in n-Si by electrochemical etching in 1972 [11] and its formation mechanism has been studied since the 1990s [12]. It shows that holes play an important role in the pore formation process and can be produced by illumination in n-Si and then collected by the pore tips to dissolve the silicon. The pore walls are depleted of holes and would be passivated against dissolution. However, holes are the majority carriers for p-Si and the pore wall would be easy to be damaged, so the macropore formation mechanism of n-type silicon is not suitable.

Macropore fabrication in p-Si was first reported by Propst and Kohl [13] in the mid-1990s using an anhydrous and HF-acetonitrile electrolyte, and then several theoretical models have been reported to explain the formation mechanism [14,15,16,17,18,19,20,21,22,23]. The first one was proposed by Lehmann and Rönnebeck [14] based on charge-transfer mechanisms across the Schottky barrier. The barrier width would influence the pore wall thickness, which needs to be smaller than double the barrier width. The next model is based on the linear stability analysis model and was proposed to describe the initial stage of macropore growth [16,17,21], but it could not properly explain the overall pore growth. In addition, the current burst model (CBM) can account for all processes of the reactive Si–electrolyte interface [18,22,23]. However, it is too general and could not predict the change in pore diameter, pore wall thickness, pore formation rate and other parameters. An autocatalytic mechanism was also proposed to understand the pore formation based on a pure chemical process [19,20]. However, there are still some experimental results that could not be explained by the existing models and mechanisms, because macropore fabrication is a complicated process including mixed effects of electronic, fluidic and chemical factors.

To make straight, smooth and highly ordered macropore arrays in p-Si, a crucial point is to promote the dissolution of the pore tips and suppress the dissolution of the pore walls. A wide range of parameters needs to be considered: crystal orientation [18], doping concentration in silicon [24], etching voltage/current [25,26], electrolyte composition [27,28,29], surface patterning [30,31,32], etc. Of these, doping concentration, also known as wafer resistivity, is an important parameter in the process of porosification. It is usually recognized that the pore diameter and wall thickness would increase with the wafer resistivity [14,30,33,34,35,36,37], and the pore density would decrease [10,24,38]. As for the etching rate, there are different opinions. Some found it would also decrease with the resistivity, when the resistivity is less than 0.02 Ω·cm [24] or larger than 1000 Ω·cm [38]. Meanwhile, Vyatkin [30] also reported an increase in the etching rate with the wafer resistivity, which is from 6 to 23 Ω·cm and larger than 1000 Ω·cm. These phenomena are mostly based on random macropore formation, and few works focus on the ordered macropore formation, including the influence mechanism. In this work, the dependence of the morphology of macropore arrays on wafer resistivity, etching voltage and time was investigated to obtain macropore arrays with a high aspect ratio (>20). Highly ordered macropore arrays were obtained by double-tank etching, and the influence mechanism of silicon wafer resistivity and the process parameters on pore morphology were discussed.

## 2. Materials and Methods

The starting materials were p-Si wafers (boron doped) with different resistivities: 1–10 Ω·cm, 10–20 Ω·cm, 20–50 Ω·cm, 50–80 Ω·cm and 100–200 Ω·cm, and with a 200 nm thick silicon dioxide deposited by low-pressure chemical vapor deposition on the surface, provided by Suzhou RDMICRO. The electrochemical etching electrolyte of the anode was composed of hydrofluoric acid (HF, ≥40%), deionized water (DIW), N, N-Dimethylformamide (DMF), Triton X-100 and hydrochloric acid (HCl, 36~38%). The conductive electrolyte of the cathode was a mixture of sodium chloride and DIW. DIW was purchased from Hongkou Baoxing deionized water factory (Shanghai, China) and other reagents were from Sinopharm Chemical Reagent Co., Ltd. (Shanghai, China).

**Figure 1:**
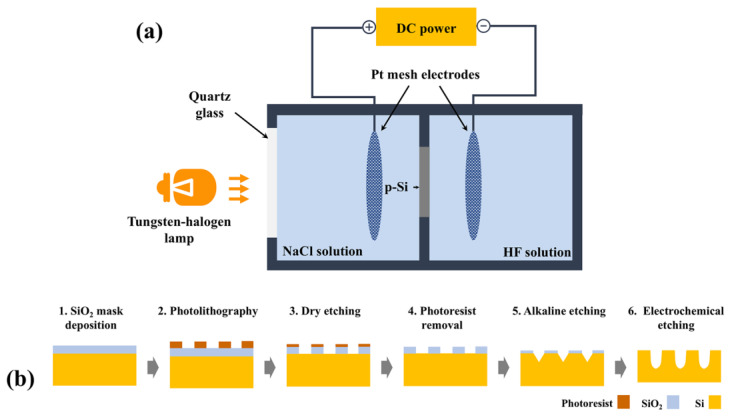
(**a**) Schematic diagram of double-tank electrochemical cell. (**b**) Schematic steps for ordered macropore fabrication process.

Highly ordered pore arrays were fabricated by applying a multistage process including photolithographic patterning and subsequent alkaline etching, as shown in Figure 1b. The patterns were a two-dimensional tetragonal lattice of squares with 3 × 3 μm and the spacing of the pits was 5 μm. The detailed fabrication process was described in the previous literature [39]. The double-tank electrochemical etching was used, as shown in Figure 1a. The etching electrolyte was an HF solution (HF: DIW: DMF = 1: 4: 5) with additives (30 mL Triton-X and 60 mL HCl), and it was agitated by stirring with a rotation speed of 200 rpm during the etching process. The conductive electrolyte was a saturated NaCl solution, circulated by a pump. The potentiostatic method was used in our experiment, and the constant voltages were set from 4 to 6 V, supplied by the CHI660c electrochemical workstation (Shanghai Chenhua Co., Shanghai, China). The backside of the silicon was illuminated by a 1000 W tungsten–halogen lamp because it requires illumination to generate minority carriers (electrons in p-Si) to participate in the cathodic reaction in double-tank electrochemical etching, leading to current transmission. The etching time was controlled from 0 to 240 min. The samples were dried by N_2_ after etching and then were cleaved for the investigation of the surface and cross-sectional morphology by a Phenom Pro scanning electron microscope (SEM, Phenom World, Eindhoven, the Netherlands).

## 3. Results and Discussion

### 3.1. Effect of Silicon Resistivity

**Figure 2:**
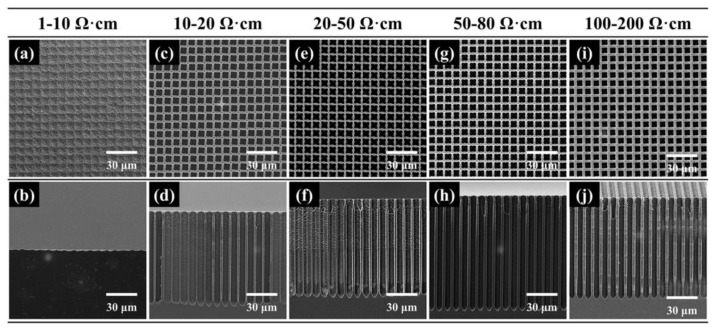
SEM images of macropore arrays fabricated on the p-type silicon with the resistivity of (**a**,**b**) 1–10 Ω·cm, (**c**,**d**) 10–20 Ω·cm, (**e**,**f**) 20–50 Ω·cm, (**g**,**h**) 50–80 Ω·cm and (**i**,**j**) 100–200 Ω·cm. All samples were etched at 5 V for 120 min.

**Figure 3:**
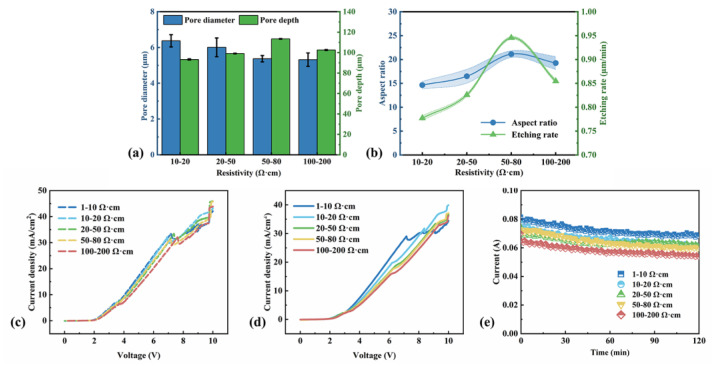
(**a**) Pore diameter and depth vs. the resistivity of p-Si. (**b**) The aspect ratio and etching rate vs. the resistivity of p-Si. All curves of etching rate mean the vertical etching rate, and the etching rate equals the pore depth divided by the total etching time. (**c**) The j-V curves of p-Si with different resistivities at the initial stage of the etching process. (**d**) The j-V curves of p-Si with different resistivity after two-hour etching. (**e**) I-t curves of macropore fabrication using p-Si wafers with different resistivities. The etching voltage is 5 V.

We investigated the influence of silicon resistivity on macropore formation at 5 V in 120 min. Their morphologies are shown in Figure 2 and the pore diameter and pore depth are shown in Figure 3a. With the increase in the resistivity, the pore diameter decreases and the wall thickness increases. However, the pore depth increases below 80 Ω·cm and then decreases. Yet, for 1–10 Ω·cm samples, only periodic and shallow pits can be observed in Figure 2a,b. It seems that the pore wall of the 1–10 Ω·cm sample is too thin and the macropore arrays could not keep stable during the long-time etching process. Meanwhile, the aspect ratio and etching rate are calculated in Figure 3b. The highest aspect ratio of 21 and largest etching rate of 0.95 μm/min could be obtained in ordered 50–80 Ω·cm samples.

The etching current density–voltage (j-V) curves of silicon wafers with different resistivities are measured at the initial stage of the etching and after two-hour etching, respectively (see Figure 3c,d). The scan rate is 50 mV/s and the starting voltage is about 2 V in this work. The current density increases with the increase in the applied voltage. The slope in this regime slightly decreases with the increase in wafer resistivity until the first peak, due to the increased ohmic drop. Then, there is a current peak at around 7~7.5 V for p-type silicon wafers with different resistivities, whose potentials tend to extend to more positive values with doping concentration decreasing. This is attributed to a larger potential drop in the space charge layer of the lower doped samples [40]. This peak means a change from pore formation to electropolishing, usually named the J_ps_ peak [14,41]. When J = J_ps_, the electrochemical system is in a steady-state condition; namely, diffusion of HF molecules and hole supply occur at the same rate. After two-hour electrochemical etching, the current density of the J_ps_ peak becomes distinct. In the 1–10 Ω·cm sample, the J_ps_ peak after etching is similar to the initial state but the current density slightly decreases. As for samples with higher resistivities, there is an obvious decrease in the current density and voltage of the J_ps_ peak, which implies that the ionic diffusion is limited in the deep pores [23] and J_ps_ would decrease with the pore depth. Interestingly, the macropore arrays are deeper in the 50–80 Ω·cm sample than the 100–200 Ω·cm sample, but J_ps_ is still larger. The decrease in the etching rate indicates that hole supply would dominate pore growth in the deep pores for the high resistive samples (˃100 Ω·cm).

Figure 3e exhibits the recorded current during the electrochemical etching processes. The current decreases with time and the sample resistivity. For the 1–10 Ω·cm sample, there are no stable macropore arrays after two-hour electrochemical etching, as shown in Figure 2a,b, but the decrease in the current with time is still observed. This demonstrates that macropores form in the 1–10 Ω·cm sample, but the resistivity is low to form thin pore walls that are difficult to stabilize during etching. For 10–80 Ω·cm samples, the current values are proximate despite their different pore morphologies, and the current decreases evidently when the resistivity is up to 100 Ω·cm. Thus, the current is sensitive to the change in the resistivity by order of magnitude.

### 3.2. Effect of Etching Voltage

**Figure 4:**
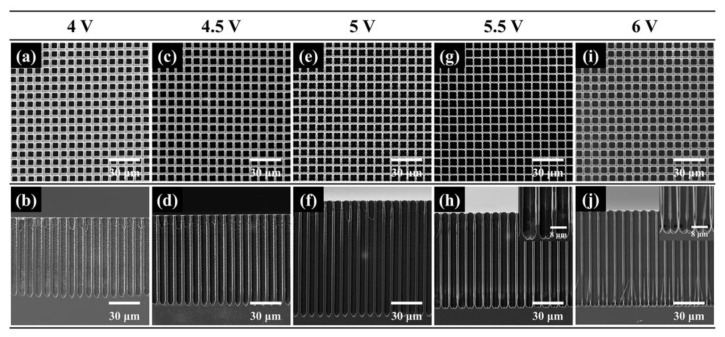
SEM images of macropore arrays fabricated on the p-type silicon with the resistivity of 50–80 Ω·cm at a voltage of (**a**,**b**) 4 V, (**c**,**d**) 4.5 V, (**e**,**f**) 5 V, (**g**,**h**) 5.5 V and (**i**,**j**) 6 V.

**Figure 5:**
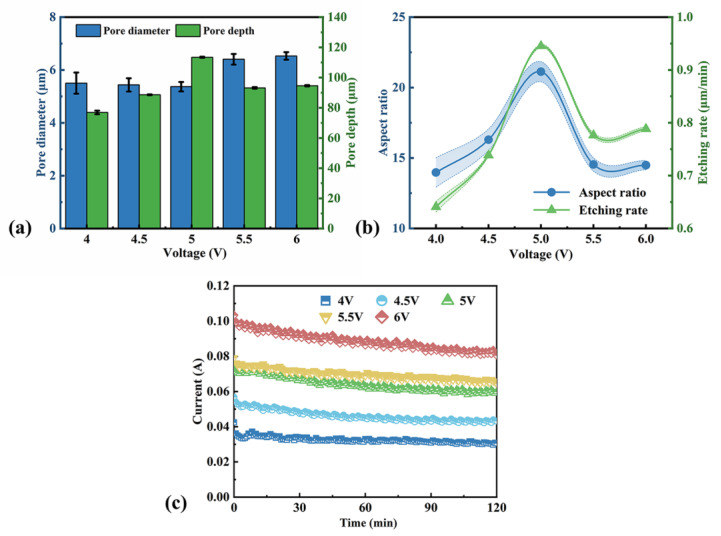
(**a**) Pore diameter and depth vs. etching voltage. (**b**) The aspect ratio vs. etching voltage. (**c**) I-t curves of macropore fabrication using p-Si wafers at different voltages.

The effect of etching voltage was also studied. The SEM images of macropore arrays obtained at different voltages are shown in Figure 4. The diameter and depth dependence of the voltage are represented in Figure 5a. When the etching voltage is less than 5 V, the pore diameter is nearly invariable, and the pore depth obviously increases with the increase in the etching voltage. When over 5 V, there is an increase in the pore diameter and a decrease in the pore depth, although the total dissolution volume increases with the etching voltage. Figure 4h,j present there are hillocks at the bottom of the pores at the etching voltage of 5.5 and 6 V. It seems plausible to infer that one pore splits into at least two smaller pores.

The aspect ratio and etching rate are calculated, as shown in Figure 5b. The optimum voltage is around 5 V. Figure 5c shows the etching current curves at different voltages during two-hour etching times, and it is seen that the current increases with the voltage and decreases slightly with time. This means high voltage can promote hole transformation to boost the etching rate. However, there is a limit on the voltage in our experiment. Above 5 V, pore splitting would occur, and it would stunt the vertical growth of pores. Figure 5c also implies the downtrend in current with time would increase with the etching voltage. The difference value of the initial current and end current is about 0.02 A for the sample etched at 6 V, and 0.004 A for the sample etched at 4 V. This is because HF diffusion in the deep pores could not match hole transportation due to high voltage, leading to a shift in the bottom reaction toward electropolishing rather than pore formation. Then, the pore sidewalls are smoother for the higher voltage etching.

**Figure 6:**
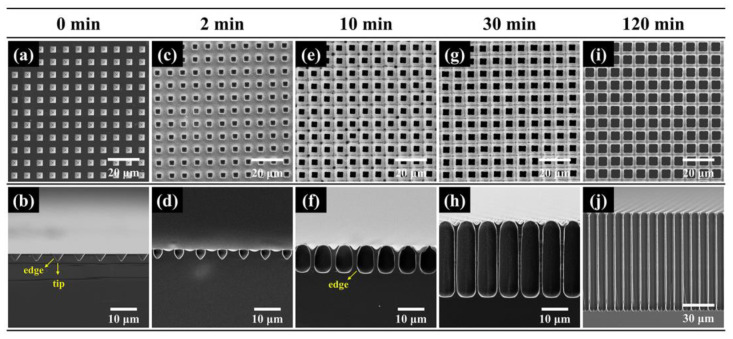
SEM images of macropore arrays etched at (**a**,**b**) 0 min, (**c**,**d**) 2 min, (**e**,**f**) 10 min, (**g**,**h**) 30 min and (**i**,**j**) 120 min. The resistivity of silicon is 50–80 Ω·cm and the etching voltage is 6 V.

To better understand the cause of pore splitting at a high voltage, pore morphologies of different times at the etching voltage of 6 V are presented in Figure 6. In 2 min, the pore bottom is still like a cone and the cone tip has the smallest radius of curvature (see Figure 6d). However, in 10 min, the pore shape changes. Due to the inequality of hole transportation and HF diffusion, electropolishing takes place in the pore bottom. This would stunt the vertical growth of the macropore, especially at the pore tips, but the horizontal growth is hardly affected. Then, the cone-shaped pores would turn into cylinder-shaped pores with the pore tips flatting out, as shown in Figure 6f. In 30 min, there is pore splitting in the cylinder-shaped pores in Figure 6h. This is due to the fact that the radius of curvature is minimized at the bottom edge, which would bring about several active sites for pore growth and cause pore splitting.

### 3.3. Effect of Etching Time

**Figure 7:**
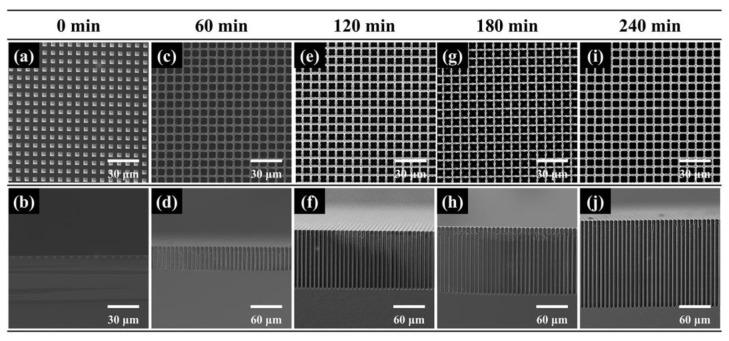
SEM images of macropore arrays etched at (**a**,**b**) 0 min, (**c**,**d**) 60 min, (**e**,**f**) 120 min, (**g**,**h**) 180 min and (**i**,**j**) 240 min. The resistivity of silicon is 50–80 Ω·cm and the etching voltage is 5 V.

**Figure 8:**
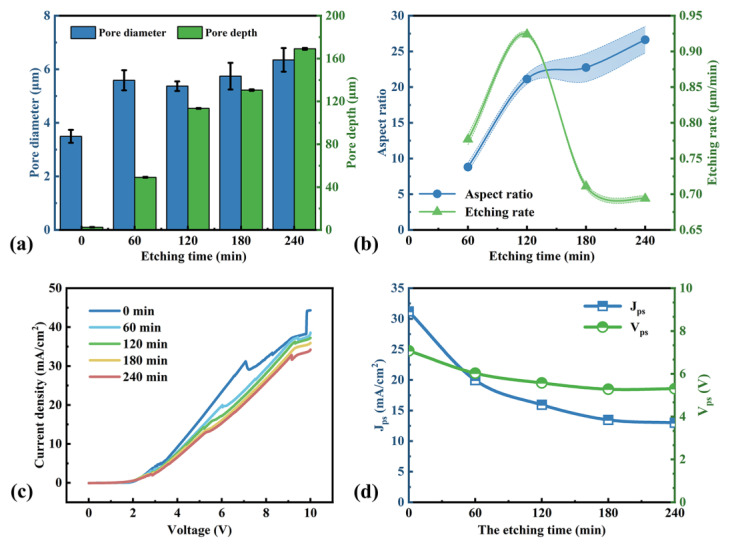
(**a**) Pore diameter and depth vs. etching time. (**b**) The aspect ratio and etching rate vs. etching time. (**c**) The j-V curves of p-Si at different etching times. (**d**) The values of J_ps_ and V_ps_ at different etching times.

Figure 7 exhibits surface and cross-sectional SEM images of samples obtained at different etching times. It seems that pore walls at the bottom are smoother than at the top. As the pore depth increases, the rate of HF diffusion into the bottom would decrease, and electropolishing that consumes more holes would occur at the bottom. Meanwhile, HF diffusion would not be limited at the top and electropolishing hardly occurs. Therefore, pore walls at the bottom would be smoother. In addition, the pore walls would be smoother with time also due to limited HF diffusion. Their pore diameters and depths are shown in Figure 8a. The diameter and depth increase with the increase in etching time. In Figure 8b, it can be seen that the highest aspect ratio (around 27) can be obtained when the etching time is 240 min. However, when the etching time is over 120 min, there is a sharp decrease in the etching rate. This is also because that deep pore is unfavorable for the movement of gas bubbles and products of silicon dissolution and the propagation of fresh electrolyte portions, especially when the pore depth is over approximately 100 μm.

The j-V curves after different etching times are exhibited in Figure 8, and their J_ps_ and V_ps_ are recorded in Figure 8d. The value of J_ps_ and V_ps_ decreases with the etching time and hardly changes after 180 min, and the current peak becomes smoothed out or even disappears. This is due to an increase in electrolyte resistivity, caused by a decrease in mobile ion concentration. The current is usually largest at the pore tip and decreases from the pore tip to the pore wall, because the curvature radius increases [42]. The first current peak, J_ps_, represents the maximum current for pore formation. When J_ps_ decreases, the gap of dissolution rate between the tip and the wall would also decrease, leading to the vertical etching rate decreasing and horizontal etching increasing. Additionally, there is another peak in Figure 8c, which corresponds to the formation of a dense oxide of low defect density [43]. If the time is extended and the voltage remains, the vertical etching rate would decrease, as in Figure 8b, and the horizontal etching would dominate during pore formation.

### 3.4. Mechanism Analysis for Electrochemical Etching

#### 3.4.1. Electrochemical Dissolution Valence

There are two different and competing reaction paths of silicon dissolution in HF solution: direct dissolution and indirect dissolution. The direct dissolution can proceed if only holes are supplied and it can make the most efficient use of the holes. It would dominate at a relatively low current range, with the following stoichiometry [44]:

$$Si+4{HF}_{2}^{-}+2h^{+}\to {SiF}_{6}^{2-}+2HF+H_{2}↑$$

The indirect dissolution needs four holes to oxidize silicon and then oxides would be dissolved by the etching solution through a purely chemical process. Due to the larger hole consumption, the indirect dissolution would dominate at a higher etching current density than the direct dissolution, as follows [44]:

$$Si+2H_{2}O+4h^{+}\to SiO_{2}+4H^{+}$$

$$SiO_{2}+6HF\to H_{2}SiF_{6}+2H_{2}O$$

We have calculated the dissolution valence in different experimental conditions in Table 1, using [41]

(1) $$n=\frac{Itm_{Si}}{e\rho_{Si}V_{D}}$$

$m_{Si}$ is the mass of a Si atom, e is the elementary charge, $\rho_{Si}$ is the density of Si and $V_{D}$ is the dissolved volume. The results show that dissolving one silicon atom needs more than two holes, which means the direct dissolution and the indirect dissolution exist together. The indirect dissolution reaction rate would increase with the resistivity and voltage. Thin pore walls may be easy to be destroyed and cause a calculation bias in the dissolved volume, such as for 1–10 Ω·cm samples. Additionally, for different etching times, the dissolution valence of a two-hour etching is largest, due to the fastest reaction rate probably.

**Table 1:** Calculated dissolution valence of samples.

Resistivity/Ω	Valence	Voltage/V	Valence	Etching Time/Min	Valence
1–10	1.83	4	2.07	60	2.60
10–20	2.60	4.5	2.63	120	2.92
20–50	2.68	5	2.92	180	2.60
50–80	2.92	5.5	2.71	240	2.71
100–200	2.97	6	3.27		

#### 3.4.2. Influence Mechanism on the Morphology of Macropores

With pore development, there is a distribution of the current density along the pore as discussed above. At the pore tips, the indirect dissolution reaction would dominate and, at the pore walls, the direct dissolution reaction would dominate. Therefore, there is a distribution of the kind of reactions and the oxide film with varying thickness along the pore bottom. The oxide film is thickest at the pore tip, but it needs some time to be dissolved. Before further propagation of the pore tip, the dissolution of the pore wall would occur, causing the reactant dilution. Therefore, it is important to restrain the dissolution reaction of the pore wall.

The condition of the pore wall passivation is that holes are depleted between two pores, which means the wall thickness needs to be less than or equal to two times the space charge region width [14]. The space charge region width is usually described by [45,46]

(2) $$SCRL=\sqrt{\frac{2\varepsilon_{r}\varepsilon_{0}\left(V_{b}-V_{a}\right)}{eN_{A}}}$$

where $\varepsilon_{r}$ = 11.9 is the relative dielectric constant of silicon, $\varepsilon_{0}$ = 8.854 × 10−12 F/m is the vacuum dielectric constant and e = 1.6 × 10−19 C is the elementary charge. Doping concentration $N_{A}$ is from 1.50 × 1016 cm−3 to 6.65 × 1013 cm−3 for 1–200 Ω·cm p-type silicon. $V_{a}$ is the applied potential on the silicon wafer during etching. $V_{b}$ is the built-in potential and can be calculated from [45]

(3) $$V_{b}=\frac{E_{g}}{2e}+\frac{kT}{e}ln\left(\frac{N_{A}}{n_{i}}\right)$$

where $E_{g}$ = 1.12 eV is the bandgap of silicon, k = 1.38 × 10−23 J/K is the Boltzmann constant, T is the absolute temperature (here 300 K) and $n_{i}$ = 1 × 1010 cm−3 is the intrinsic concentration of silicon. Based on this, two times SCRL (2SCRL) can be calculated.

**Figure 9:**
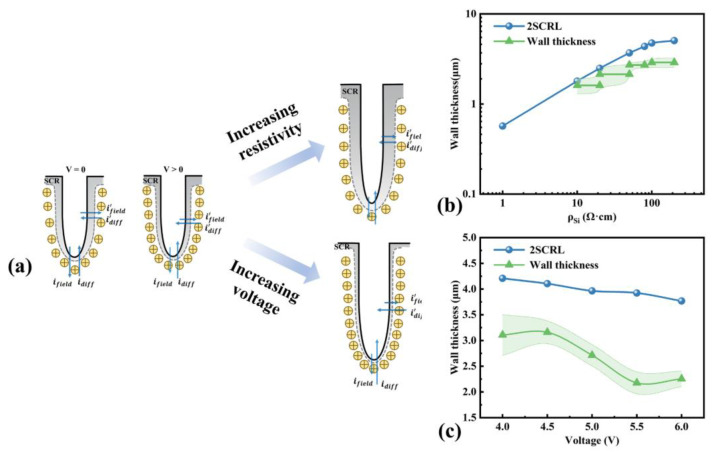
(**a**) Schematic diagram of the effect of the wafer resistivity and etching voltage on the space charge region width, diffusion current and field current. (**b**) Values of pore wall thickness from Figure 2 as a function of the silicon resistivity together with the calculated values of two times 2SCRL. (**c**) Values of pore wall thickness from Figure 4 as a function of the etching voltage together with the calculated values of two times 2SCRL.

**Figure 10:**
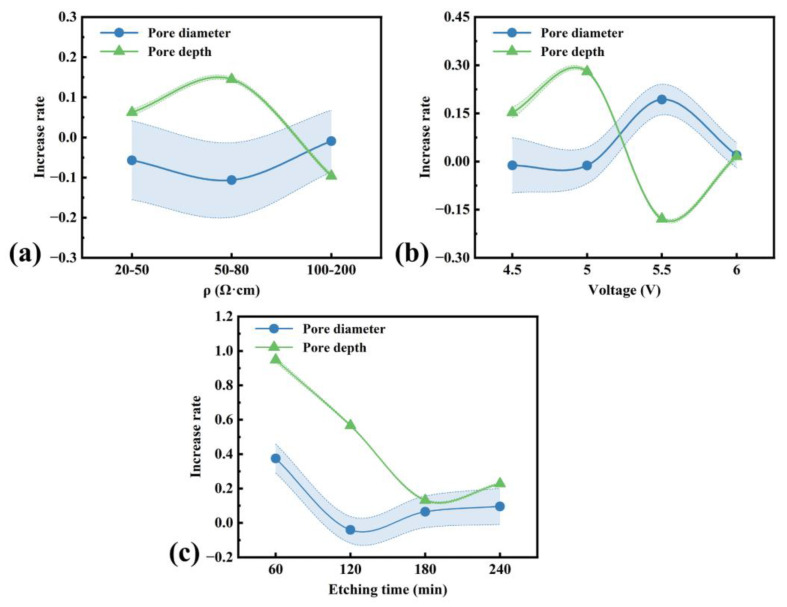
(**a**) Increase rates of pore diameters and depths for the wafer resistivity. (**b**) Increase rates of in pore diameters and depths for the etching voltage. (**c**) Increase rates of pore diameters and depths for the etching time. The increase rate equals the increase value divided by the original value.

We compared the values of 2SCRL and the wall thickness obtained from samples etched under different silicon resistivity and etching voltages, as shown in Figure 9b and Figure 9c, respectively. All the wall thicknesses are less than 2SCRL. This indicates it is not easy for holes to move into the pore wall, so the dissolution reaction rate of the pore wall is generally low, especially compared with the pore tip. This can be proved by the increase rates of pore diameters in Figure 10a,b. The increase rates of pore diameters are less than or close to 0 except when pore splitting is present.

The tendency of the wall thickness variation is the same as the change in the space charge region width, and the influence mechanism on the pore diameter is shown in Figure 9a. When silicon resistivity increases, hole concentration in the bulk silicon would decrease. They would increase the difficulty of injecting holes into the pore wall, so SCRL and the wall thickness would increase and the pore diameter would decrease (see Figure 3a and Figure 9b). Meanwhile, the electric field direction generated by the etching voltage is the same as the hole diffusion direction. When the etching voltage increases, it promotes hole diffusion into the pore wall, causing a decrease in SCRL and the wall thickness, according to Figure 9c, and the pore diameter would increase according to Figure 5a.

However, the influence on the pore depth is determined more by the relative nature of etching. Dissolution reactions of the pore tip and the pore wall are competitive during the etching process. The former would increase the pore depth and the latter would increase the pore diameter. The relative rates of these would influence the morphology of the macropore arrays. In low-doped p-type silicon, the current is usually dominated by diffusion. As mentioned above, an oxidation reaction consuming four holes would tend to occur at the pore tip, causing a maximum hole gradient and a larger diffusion current $i_{diff}$. At the thermal equilibrium and at zero voltage, the diffusion current $i_{diff}$ would be compensated by the field current $i_{field}$, and so is the pore wall. However, the diffusion current $i_{diff}^{'}$ and the field current $i_{field}^{'}$ at the pore wall would be less than them at the pore tip due to geometric field enhancement, as shown in Figure 9a.

When a forward bias is applied, the SCRL would decrease. $i_{diff}$ and $i_{diff}^{'}$ would increase, while $i_{field}$ and $i_{field}^{'}\;$ would decrease. The total current at the pore tip $i_{tip}=i_{diff}-i_{field}$ and at the pore wall $i_{wall}^{'}=i_{diff}^{'}-i_{field}^{'}$ would increase with the etching voltage. However, $i_{tip}$ would be always larger than $i_{wall}^{'}$ due to the smaller curvature radius. Therefore, increasing the etching voltage would mainly increase the tip current to strengthen the pore growth rate. According to Figure 10b, the increase rate of the pore depth is larger than 0 before 5 V, while the increase rate of the pore diameter is less than 0. Further, the differential current between $i_{tip}$ and $i_{wall}^{'}$ would increase with the etching voltage. It may cause the increasing difference of the increase rate between the pore depth and the pore diameter before 5 V. It is worth mentioning that one pore will split into several pores when the voltage is too high (about 5.5 V in our work). The wall dissolution in the several pores would need more holes. It may cause a sharp decrease in the increase rate of the pore depth and an obvious increase in the increase rate of the pore diameter, and the increase in the pore diameter would dominate. This is not profitable for obtaining a high aspect ratio and deep macropores.

When the silicon resistivity increases and the etching voltage remains unchanged, the hole supply would decrease. The diffusion current and the field current would decrease as well, while the differential current between $i_{tip}$ and $i_{wall}^{'}$ would increase at a certain range. This may lead to an increase in the pore depth and a decrease in the pore diameter, as shown in Figure 3a. Also, the difference in the increase rate between the pore depth and the pore diameter would increase, as shown in Figure 10a. However, when the silicon resistivity is larger than a certain value (about 100 Ω·cm in our work), the problem of insufficiency of hole supply would dominate the dissolution of the pore tip, causing the pore growth rate to decrease significantly, especially less than the increase rate of the pore diameter.

When the etching time increases, the etching rate would decrease due to diffusion limitation in the deep pore, as shown in Figure 8b. After some time, a diffusion rate decrease in the deep pore would limit the growth of pore depth, which may cause a sharp decrease in the increase rate of the pore depth and then tend to a slight change, as shown in Figure 10c. This is the same as the pore diameter. At 180 min, the increase rate of the pore depth and the pore diameter is close to each other. It is not helpful for a high-efficient fabrication of deep macropore arrays.

From the experimental data and the analysis, the fabrication of ordered macropore arrays in the silicon is due to preferential dissolution in the different regions. Optimizing the process and silicon wafer parameters can help us to obtain highly ordered macropore arrays by changing the relative dissolution nature between the pore walls and the pore tips.

## 4. Conclusions

The influence of silicon resistivity, etching voltage and etching time on pore morphology was studied. The mechanism for electrochemical etching of silicon under different etching conditions was explained based on the analysis of the relative dissolution nature of the different regions. Increasing silicon resistivity would decrease the pore diameter due to the increase in the space charge region width. The maximum pore depth is about 113.44 μm in the 50–80 Ω·cm sample, because a further decrease in hole supply would decrease the dissolution reaction rate at the pore tips. Increasing the etching voltage would promote hole diffusion to obtain a high etching current and rate at a certain range, leading to an increase in the pore depth and diameter. However, too high voltage (over 5 V in our experiment) would cause pore splitting due to the change in the pore morphology in the pore bottom. The aspect ratio of 21 and the maximum etching rate of 0.92 μm/min can be obtained in 120 min for the 50–80 Ω·cm sample at the etching voltage of 5 V. The electrochemical etching process presented in this work could be applied in the preparation of three-dimensional devices like silicon capacitors.

## Data Availability

All the relevant data are included in this published article.

## References

[1] Rashid R.B., Alwan A.M., Mohammed M.S. (2023). Improved Difenoconazole Pesticide Detection Limit via Double-Sided Porous Silicon Layers’ Electrical Sensor. Mater. Chem. Phys..

[2] Gozeh B.A. (2023). Fast-Response PN-Photodetector Based on V_2_O_5_-Nanorods/Macroporous Si Heterojunction. Thin Solid Films.

[3] Garzon-Roman A., Zuñiga-Islas C., Cuate-Gomez H.D., Heredia-Jimenez A. (2023). TiO_2_/Porous Silicon Heterostructures Formation by Simple and Low-Cost Methods for Electronics Applications. Sens. Actuator A Phys..

[4] Sun Z., Gu M., Li Q., Liu X., Liu B., Zhang J., Huang S., Ni C. (2021). Performance of a CsI(Tl) Scintillation Screen with a Dual-Periodic Structure Based on an Oxidized Silicon Micropore Array Template in X-Ray Imaging. Opt. Express.

[5] Birner A., Wehrspohn R.B., Gösele U.M., Busch K. (2001). Silicon-Based Photonic Crystals. Adv. Mater..

[6] Zavatski S., Dubkov S., Gromov D., Bandarenka H. (2023). Comparative Study of SERS-Spectra of NQ21 Peptide on Silver Particles and in Gold-Coated “Nanovoids”. Biosensors.

[7] Strambini L., Paghi A., Mariani S., Sood A., Kalliomäki J., Järvinen P., Toia F., Scurati M., Morelli M., Lamperti A. (2020). Three-Dimensional Silicon-Integrated Capacitor with Unprecedented Areal Capacitance for on-Chip Energy Storage. Nano Energy.

[8] Jhajhria D., Tiwari P., Chandra R. (2023). Passivation of Macroporous Si Using Sputtered TiN Coating for On-Chip Energy Storage. J. Power Sources.

[9] Maiolo J.R., Atwater H.A., Lewis N.S. (2008). Macroporous Silicon as a Model for Silicon Wire Array Solar Cells. J. Phys. Chem. C.

[10] Korotcenkov G., Cho B.K. (2010). Silicon Porosification: State of the Art. Crit. Rev. Solid State Mat. Sci..

[11] Theunissen M.J.J. (1972). Etch Channel Formation during Anodic Dissolution of N-Type Silicon in Aqueous Hydrofluoric Acid. J. Electrochem. Soc..

[12] Lehmann V. (1993). The Physics of Macropore Formation in Low Doped N-Type Silicon. J. Electrochem. Soc..

[13] Propst E.K., Kohl P.A. (1994). The Electrochemical Oxidation of Silicon and Formation of Porous Silicon in Acetonitrile. J. Electrochem. Soc..

[14] Lehmann V., Ronnebeck S. (1999). The Physics of Macropore Formation in Low-Doped P-Type Silicon. J. Electrochem. Soc..

[15] Kang Y., Jorné J. (1997). Dissolution Mechanism for P-Si During Porous Silicon Formation. J. Electrochem. Soc..

[16] Valance A. (1997). Theoretical Model for Early Stages of Porous Silicon Formation from N- and P-Type Silicon Substrates. Phys. Rev. B.

[17] Chazalviel J.N., Wehrspohn R.B., Ozanam F. (2000). Electrochemical Preparation of Porous Semiconductors: From Phenomenology to Understanding. Mater. Sci. Eng. B.

[18] Christophersen M., Carstensen J., Rönnebeck S., Jäger C., Jäger W., Föll H. (2001). Crystal Orientation Dependence and Anisotropic Properties of Macropore Formation of P- and N-Type Silicon. J. Electrochem. Soc..

[19] Corbett J.W., Shereshevskii D.I., Verner I.V. (1995). Changes in the Creation of Point Defects Related to the Formation of Porous Silicon. Phys. Status Solidi A.

[20] Kooij E.S., Vanmaekelbergh D. (1997). Catalysis and Pore Initiation in the Anodic Dissolution of Silicon in HF. J. Electrochem. Soc..

[21] Kang Y., Jorné J. (1993). Morphological Stability Analysis of Porous Silicon Formation. J. Electrochem. Soc..

[22] Carstensen J., Christophersen M., Hasse G., Föll H. (2000). Parameter Dependence of Pore Formation in Silicon within a Model of Local Current Bursts. Phys. Status Solidi A.

[23] Föll H., Christophersen M., Carstensen J., Hasse G. (2002). Formation and Application of Porous Silicon. Mater. Sci. Eng. B.

[24] Martín-Sánchez D., Ponce-Alcántara S., Martínez-Pérez P., García-Rupérez J. (2019). Macropore Formation and Pore Morphology Characterization of Heavily Doped P-Type Porous Silicon. J. Electrochem. Soc..

[25] Cozzi C., Polito G., Strambini L.M., Barillaro G. (2018). High Anodic-Voltage Focusing of Charge Carriers in Silicon Enables the Etching of Regularly-Arranged Submicrometer Pores at High Density and High Aspect-Ratio. Front. Chem..

[26] Zhang L., Gao K., Zeng Z., Wang K., Zhao C., Ge D., Zhang L. (2023). Controlled and Fast Fabrication for P-Type Porous Silicon Structures with a High Aspect Ratio by Electrochemical Etching. J. Electron. Mater..

[27] Cozzi C., Polito G., Kolasinski K.W., Barillaro G. (2017). Controlled Microfabrication of High-Aspect-Ratio Structures in Silicon at the Highest Etching Rates: The Role of H_2_O_2_ in the Anodic Dissolution of Silicon in Acidic Electrolytes. Adv. Funct. Mater..

[28] Defforge T., Diatta M., Valente D., Tran-Van F., Gautier G. (2013). Role of Electrolyte Additives during Electrochemical Etching of Macropore Arrays in Low-Doped Silicon. J. Electrochem. Soc..

[29] Ossei-Wusu E., Carstensen J., Quiroga-González E., Amirmaleki M., Föll H. (2013). The Role of Polyethylene Glycol in Pore Diameter Modulation in Depth in P-Type Silicon. ECS J. Solid State Sci. Technol..

[30] Vyatkin A., Starkov V., Tzeitlin V., Presting H., Konle J., König U. (2002). Random and Ordered Macropore Formation in P-Type Silicon. J. Electrochem. Soc..

[31] Kobayashi K., Harraz F.A., Izuo S., Sakka T., Ogata Y.H. (2007). Macropore Growth in a Prepatterned P-Type Silicon Wafer. Phys. Status Solidi A.

[32] Xiao-Qing B., Dao-Han G., Ji-Wei J. (2008). Observation of a Diverse Deviation from Macropore-Formation Theory in Silicon Electrochemistry. Chin. Phys. B.

[33] Chazalviel J.N., Ozanam F., Gabouze N., Fellah S., Wehrspohn R.B. (2002). Quantitative Analysis of the Morphology of Macropores on Low-Doped p-Si: Minimum Resistivity. J. Electrochem. Soc..

[34] Hamm D., Sakka T., Ogata Y.H. (2003). Porous Silicon Formation under Constant Anodization Conditions: Homogeneous Regime or Transition?. J. Electrochem. Soc..

[35] Zegrya G.G., Ulin V.P., Zegrya A.G., Freiman V.M., Ulin N.V., Fadeev D.V., Savenkov G.G. (2023). Limiting Thickness of Pore Walls Formed in Processes of Anode Etching of Heavily Doped Semiconductors. Tech. Phys..

[36] Lust S., Lévy-Clément C. (2000). Macropore Formation on Medium Doped p-Type Silicon. Phys. Status Solidi A.

[37] Harraz F.A., Kamada K., Kobayashi K., Sakka T., Ogata Y.H. (2005). Random Macropore Formation in P-Type Silicon in HF-Containing Organic Solutions: Host Matrix for Metal Deposition. J. Electrochem. Soc..

[38] Wehrspohn R.B., Chazalviel J.-N., Ozanam F. (1998). Macropore Formation in Highly Resistive p-Type Crystalline Silicon. J. Electrochem. Soc..

[39] Zhang J., Zhang F., Ma M., Liu Z. (2024). Fabrication of Ordered Macropore Arrays in N-Type Silicon Wafer by Anodic Etching Using Double-Tank Electrochemical Cell. Micromachines.

[40] Zhang X.G. (2001). Electrochemistry of Silicon and Its Oxide.

[41] Lehmann V., Föll H. (1990). Formation Mechanism and Properties of Electrochemically Etched Trenches in N-Type Silicon. J. Electrochem. Soc..

[42] Zhang X.G. (1991). Mechanism of Pore Formation on n-Type Silicon. J. Electrochem. Soc..

[43] Lehmann V. (2002). Electrochemistry of Silicon: Instrumentation, Science, Materials and Applications.

[44] Surdo S., Barillaro G. (2024). Voltage- and Metal-Assisted Chemical Etching of Micro and Nano Structures in Silicon: A Comprehensive Review. Small.

[45] Pierret R.F. (1996). Semiconductor Device Fundamentals.

[46] Keshavarzi S., Mescheder U., Reinecke H. (2018). Formation Mechanisms of Self-Organized Needles in Porous Silicon Based Needle-Like Surfaces. J. Electrochem. Soc..

